# Cough in Pulmonary Phthisis

**Published:** 1903-09

**Authors:** J. Leffingwell Hatch

**Affiliations:** B. Sc., M. D., F. R. M. S., London


### Cough in Pulmonary Phthisis.

BY J. LEFFINGWELL HATCH, B. SC., M. D., F. R. M S., LONDON.

As broods silence back of sound, so also stands designer back of design, and the logical mind of man has ever thus traced a presumptive relation between the thing observed and its supposed origin, and called them respectively cause and effect.

Thus in medicine we look from symptoms to a cause, and if post-mortem we find a definite lesion we too often jump to the conclusion that it must be the very thing we are looking for, and are apt to forget that back of this change of structure lingers the first real cause in perverted physiologic function.

One of the best known and oldest symptoms, and one which occurs from diverse causes, is cough, and this, with another, almost as common and well known, dyspnea, go hand in hand among the various affections of the respiratory organs.

In pulmonary phthisis cough is usually the first symptom manifest and lasts throughout the disease, but the cause is not the same in each stage and consequently requires careful study and varying treatment in the different stages.

The earliest physiologic alteration is a hyperemia usually occurring at the apices. This congestion of the capillaries is the causal irritation that brings about the cough reflexly through the medium of the nervous system. Here a nerve depressant and vaso-motor dilator is indicated rather than an analgesic and expectorant.

In the next stage of consolidation the hepatized tissue acts as a foreign body and likewise reflexly brings about a useless cough in the vain effort to get rid of itself. In this stage resolution should be established by means of an alterative and the nerves quieted by a sedative.

In the third stage where the tissue has undergone cheesy degeneration and broken down, it really is a foreign body that causes the cough, which can only be relieved by its removal, hence we give stimulating expectorants in combination with sedatives and analgesics to relieve the nervous spasms and consequent pain.

The sum total of the forces of a consumptive is at the most a low figure, and we try to keep this up by a high diet that often deranges other organs, whereas regard to the conservation of force by lessening the cough will give the same result without detriment to other emunctories.

To allay cough, then, has been the aim of therapeutics from time immemorial, and of the different concoctions and mixtures that have been vaunted and foisted upon long-suffering humanity their name is legion.

Probably the greatest boon that ever came to us in the form of medicine is opium, and some form or other of this drug has been and always will be used to a great extent as an ingredient in every cough mixture.

Of the alkaloids of opium, morphia has probably been the most popular until recent years, when codein has claimed considerable attention and threatened to usurp its place; but since the discovery of heroin, by Prof. H. Dresser, of Elberfeld, Germany, in 1898, this has been made impossible, and the new analgesic after careful study both in Europe and America has found great favor among practitioners, especially in diseases of the respiratory organs.

In the fall of 1900 my attention was called to Glyco-Heroin

(Smith), and I tried a sample bottle on a patient with such gratifying results that I determined to make further observations.

What these results were, the clinical record below tells more graphically than worded phrases of description could hope to do.

It does not nauseate, and can be given in teaspoonful doses as often as every two hours to adults, dose of course being graduated in children according to the age, although they tolerate heroin where opium would produce untoward results.

The greatest advantage this preparation has over all others lies in the fact that it does not contain anything that deranges the stomach, and can be given indefinitely without the patient turning against it.

The majority of cough mixtures contain sugar, which is bound to undergo more or less fermentation; opium, which constipates and affects respiration, and belladonna, which checks the secretions, so that if they are able to lull the patient into oblivion of his condition for a few hours on account of the large amount of narcotic they contain, he awakes to find a stagnation of secretions with renewed paroxysms of coughing, and "pushing the mustard to fanaticism" for further relief he eventually becomes a slave to opium.

I have used Glyco-Heroin (Smith) now in fifty cases, with the unvarying result that it relieved the cough, reduced the temperature, increased the volume of respiration, and allayed the night sweats, while at the same time it did not derange the stomach or cause constipation, did not produce vertigo nor nausea, never weakened the respiration, nor caused deleterious effects upon the heart, so that I can frankly say that without doubt we have in this compound the ideal cough mixture for the cough of phthisis pulmonalis.

The cases that I here quote I have selected from a series of fifty-three, with the idea of not citing cases so near alike as to produce monotonous repetition, no matter how gratifying the results.

As has been well said of this preparation, it is not only a true pharmaceutical product but an ethical one as well, and one that the physician can use understandingly, as its composition and physiologic action are well known.

Unfortunately all good things are sooner or later imitated, and something put forward as just as good but cheaper, and Glyco-Heroin (Smith) is no exception to this rule, so if results are not satisfactory, substitution must be at the bottom of it.

